# AV block after flutter ablations?

**DOI:** 10.1007/s12471-014-0560-x

**Published:** 2014-05-08

**Authors:** A. W. G. J. Oomen, L. R. C. Dekker, A. Meijer

**Affiliations:** 1 Department of Cardiology, Catharina Ziekenhuis, Michelangelolaan 2, Eindhoven, 5623 EJ the Netherlands; 2 Catharina Hospital, Michelangelolaan, Eindhoven, 5623 EJ the Netherlands

## Rhythm Puzzle-Question

A 68-year-old, otherwise healthy male, presented at the emergency department with dizziness and near-syncope 4 days after a redo catheter ablation procedure for atypical right atrial flutter.

In 1980 he underwent surgical closure of an atrial septal defect. One year prior to the current problem, a catheter ablation procedure in the right atrium was performed because of an atypical isthmus-dependent atrial flutter. During this procedure the cavotricuspid isthmus was blocked. Four days before presentation at the emergency department, a redo procedure was performed because of another postincisional atrial flutter. This time the flutter circuit was located in the septum and was successfully ablated. The earlier ablation lines were well recognisable and still formed block.

Physical examination and laboratory tests did not demonstrate any abnormalities.

**Fig. 1:**
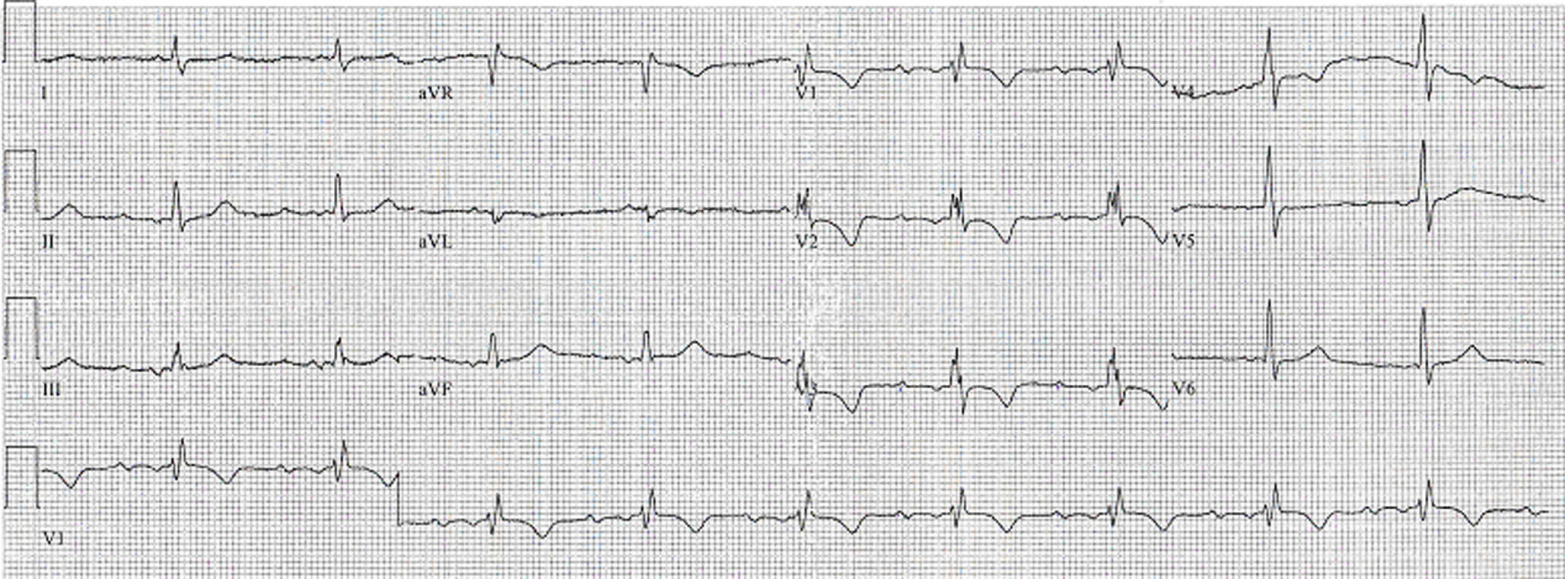
Electrocardiogram on admission

**Fig. 2:**
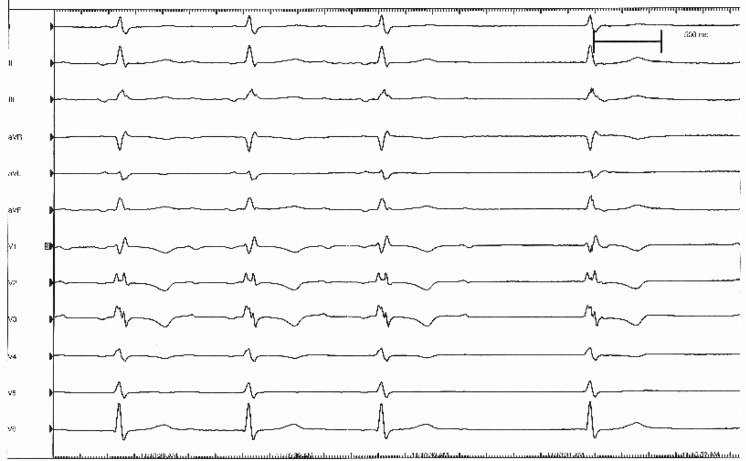
Rhythm recording in cathlab with paper speed 50 mm/s

A 12-lead electrocardiogram and rhythm recording after his re-admission for near-syncope are shown in Fig. 1 and 2 respectively. What is your diagnosis and what is the mechanism?

